# Superior Vena Cava Approach for Farapulse Pulsed-Field Ablation in Patient with Paroxysmal Atrial Fibrillation: A Case Report

**DOI:** 10.3390/jcdd12120477

**Published:** 2025-12-04

**Authors:** Qitong Zhang, Linhua Kuang, Xiaoyu Wu, Zikan Zhong, Shaowen Liu, Genqing Zhou

**Affiliations:** 1 Department of Cardiology, Shanghai General Hospital, Shanghai Jiao Tong University School of Medicine, No. 100, Haining Road, Shanghai 200080, China; qitong_zhang@sjtu.edu.cn (Q.Z.); kuanglh@163.com (L.K.); xiaoyu.wu@shgh.cn (X.W.); zzk1226@sjtu.edu.cn (Z.Z.); shaowenliu@sjtu.edu.cn (S.L.); 2 Department of Cardiology, Ji’an Central People’s Hospital, Ji’an 343000, China

**Keywords:** atrial fibrillation, superior vena cava approach, pulsed-field ablation, ICE-guided transseptal puncture

## Abstract

**Background**: Catheter ablation of atrial fibrillation (AF) is now a Class I recommendation therapy. However, the standard inferior vena cava (IVC) approach of catheter ablation is not feasible in all patients. **Case presentation**: We report a case of a 64-year-old woman in whom guidewire passage was hindered by prior left iliac vein stent placement and with symptomatic recurrent paroxysmal AF who underwent successful pulmonary vein isolation with a pulsed-field ablation system by superior vena cava (SVC) access from the right internal jugular vein. **Conclusions**: PFA administered via the SVC provides an effective and efficient treatment strategy for patients with paroxysmal AF ineligible for standard IVC catheter ablation.

## 1. Introduction

According to the 2023 guidelines for the diagnosis and management of atrial fibrillation (AF), catheter ablation is a Class I (Level of Evidence A) recommendation for patients with symptomatic, drug-refractory paroxysmal AF (PAF) [1]. Durable pulmonary vein isolation (PVI) is the cornerstone of AF catheter ablation [2]. The ablation procedure is typically performed with the inferior vena cava (IVC) approach via femoral venous access. However, the standard IVC approach is not feasible in all patients.

Pulsed field ablation (PFA) has emerged as a promising innovation in AF catheter ablation, which utilizes non-thermal energy to selectively induce cardiomyocyte cell death via irreversible electroporation [3]. Compared to conventional thermal ablation techniques, PFA minimizes the risk of collateral tissue damage, such as esophageal injury and phrenic nerve injury [4]. Furthermore, studies indicate that the duration of the PFA procedure is significantly shorter than that using thermal energy [5].

We report successful PVI using the Farapulse™ PFA system via the superior vena cava (SVC) approach from the right internal jugular vein, in a PAF patient in whom guidewire passage was impaired at the iliac vein bifurcation due to a prior iliac vein stent placement, employing single transseptal puncture under general anesthesia.

## 2. Case Presentation

A 64-year-old woman with symptomatic, drug-refractory PAF for more than 10 years was referred to our hospital for catheter ablation. She had a history of hypertension and May–Thurner syndrome, also known as iliac vein compression syndrome, which is a vascular disorder characterized by compression of the left common iliac vein by the right common iliac artery and often leads to venous stasis, lower extremity edema, pain, or deep vein thrombosis (DVT). Due to severe compression of the left common iliac vein, she underwent stenting two years ago, and was maintained on edoxaban, valsartan, and metoprolol succinate.

Transoesophageal echocardiography was performed to exclude thrombus in the left atrium. An initial attempt was made to perform radiofrequency catheter ablation with conscious sedation, following standard protocols. Right femoral vein access was successfully obtained; however, the advancement of guidewires into the IVC was unsuccessful. Venography revealed that the patient’s left iliac vein stent prevented guidewire entry into the IVC (Supplementary Video S1). Then, we attempted to traverse the stent using a Radifocus^®^ Guidewire with a transseptal sheath over the wire. The guidewire failed to cross the stent. Then, we attempted to use the left femoral vein access, but it also failed. The procedure was consequently aborted and rescheduled.

**Figure 1:**
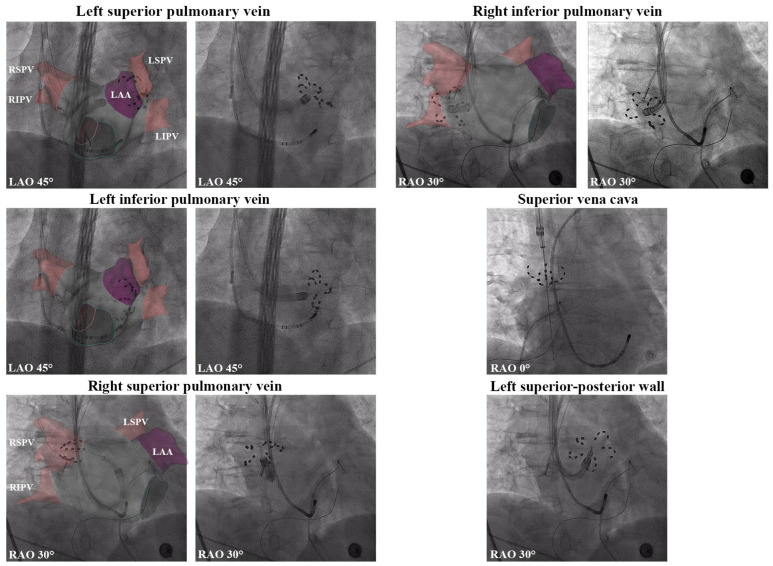
Ablation catheter in different configurations.(LSPV: Left superior pulmonary vein; LIPV: Left inferior pulmonary vein; RSPV: Right superior pulmonary vein; RIPV: Right inferior pulmonary vein; LAA: left atrial appendage).

**Figure 2:**
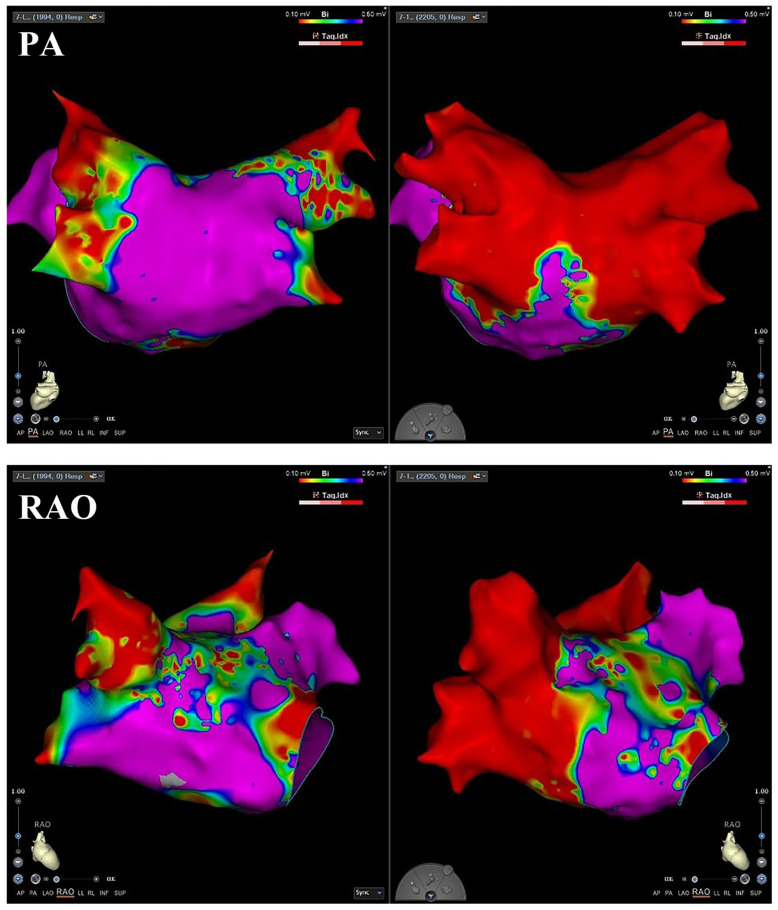
Voltage mapping pre- and post-ablation.

Computed tomography was confirmed the presence of four anatomically normal pulmonary veins. Subsequently, the procedure was reattempted under general anesthesia two days later. Access was established via the left subclavian vein, a decapolar catheter (Biosense Webster, Irvine, CA, USA) was positioned within the coronary sinus (CS), and an intracardiac echocardiography (ICE) catheter was advanced into the right atrium. Subsequent right internal jugular vein access was obtained, and an 8.5-Fr steerable sheath (Vizigo^®^; Biosense Webster) was advanced into the right atrium. After successful ICE-guided transseptal puncture (Supplementary Videos S2 and S3), electroanatomical mapping (CARTO^®^ 3 System; Biosense Webster, city, CA, USA) was performed using a multipolar mapping catheter (PentaRay^®^; Biosense Webster, Irvine, CA, USA). The Vizigo sheath was then exchanged over a guidewire for the Faradrive™ steerable sheath (Boston Scientific, MA, USA), and the FARAWAVE™ PAF catheter (Boston Scientific, Natick, MA, USA) was introduced into the left atrium. Ablation was performed by positioning the catheter sequentially at each pulmonary vein (PV) ostium and delivering pulsed electrical energy in both basket and flower configurations to achieve PVI (Figure 1). Supplementary ablation targeting the left posterior wall was also completed. Post ablation, voltage remapping of the left atrium was performed using the CARTO system to confirm lesion efficacy (Figure 2). Bidirectional conduction block across all PVs and the posterior wall was verified. Then, the PFA system was withdrawn to the right atrium, and the SVC was isolated empirically. After catheter removal, heparin anticoagulation was reversed with protamine. The right jugular vein puncture site was surgically closed, and the left subclavian vein access sites underwent manual compression for hemostasis.

The procedure time was 240 min, and the fluoroscopy time was 45 min. There were a total of 36 ablation applications, and the time from initial ablation to posterior wall isolation was 30 min. At the 6-month follow-up, no recurrence of AF was documented.

## 3. Discussion

To date, only three reported cases describe the use of the Farapulse™ system via an SVC approach [6,7]. To our knowledge, this represents the first documented case of Farapulse™ ablation performed with ICE-guided transseptal puncture via the jugular access.

For patients with an impaired IVC, AF ablation presents distinct technical challenges. In such cases, an SVC approach serves as an alternative option. However, compared to the conventional IVC route, SVC access is associated with increased risks of complications—most notably pneumothorax, as well as arterial puncture, hemothorax, mediastinal hematoma, and puncture-site hematoma. To mitigate these risks, general anesthesia represents a well-justified choice. It not only ensures patient immobility and stable operating conditions but also facilitates controlled ventilation, which may help reduce respiratory-related catheter displacement and improve procedural precision.

Transseptal puncture via an SVC approach is a critical procedural step. ICE-guided access demonstrates potential advantages over transesophageal echocardiography (TEE) in this context. CARTO system-integrated ICE provides operators with superior visualization of the interatrial septum, enabling clearer identification of the optimal puncture site compared to TEE. Additionally, TEE requires dedicated operator assistance and places an echocardiography machine near the patient’s head, which may impede catheter manipulation during ablation. Steerable sheaths are preferred for this approach due to their enhanced rigidity and maneuverability relative to non-steerable sheaths. The conventional transseptal needle is suboptimal in this setting, primarily because it lacks adaptability to patient-specific anatomy and struggles to maintain curvature stability via the SVC approach. Based on our experience with AF ablation using the SVC approach, the stiff tail of a J-shaped guidewire may serve as a more effective tool for transseptal puncture than standard needles when combined with steerable sheaths and ICE.

All ablation catheters are designed for the standard IVC approach, rendering them unsuitable for SVC access. Steering limitations in this approach prolong procedure time significantly. Compared to point-by-point thermal ablation, the one-shot PFA catheter achieves comparable efficacy while substantially reducing procedure time and left atrial (LA) dwell time. Thus, PFA provides a strategic solution to streamline ablation and enhance efficiency in AF procedures via the SVC approach.

PFA has demonstrated noninferiority to conventional thermal ablation in patients with AF. Its tissue-selective mechanism of electroporation offers a significant advantage, particularly in areas where thermal energy poses a risk of severe collateral injury to peri-atrial structures, such as the esophagus and phrenic nerve. The efficacy and safety of PFA have been validated in large-scale randomized controlled trials involving extensive patient cohorts [8,9]. Recently, the application of PFA has been extended beyond pulmonary vein isolation, with increasing exploration of its utility in extra-PV targets. In patients with atrial arrhythmias originating from the SVC, PFA enabled successful SVC isolation without causing permanent injury to adjacent critical structures such as the phrenic nerve or sinus node [10]. Additionally, PFA has been employed for mitral isthmus (MI) ablation, coronary sinus (CS) isolation, and left atrial appendage (LAA) isolation. Interestingly, while acute procedural success was observed for MI ablation, CS isolation, and LAA isolation, the rates of chronic conduction block and durable isolation remained considerably lower [11]. These findings suggest that although PFA may represent a promising and effective modality for PAF, particularly via the SVC approach, careful patient selection and thorough procedural planning are essential when considering PFA for interventions targeting extra-PV areas. A comprehensive assessment of individual anatomy and arrhythmogenic substrate, as well as judicious selection of energy, is imperative prior to the ablation.

## Data Availability

The original contributions presented in this study are included in the article/Supplementary Material. Further inquiries can be directed towards the corresponding author.

## Supplementary Materials

The following supporting information can be downloaded at https://www.mdpi.com/article/10.3390/jcdd12120477/s1, Video S1: The first attempt; Video S2: Transseptal puncture; Video S3: ICE-guided transseptal puncture.

## Abbreviations

The following abbreviations are used in this manuscript:

AF	Atrial Fibrillation
PAF	Paroxysmal AF
IVC	Inferior Vena Cava
SVC	Superior Vena Cava
PFA	Pulsed Field Ablation
DVT	Deep Vein Thrombosis
